# Precision Medicine in Diabetic Kidney Disease: A Narrative Review Framed by Lived Experience

**DOI:** 10.1177/20543581231209012

**Published:** 2023-10-30

**Authors:** Mallory L. Downie, Arlene Desjarlais, Nancy Verdin, Tania Woodlock, David Collister

**Affiliations:** 1 McGill University Health Center Research Institute, Montreal, QC, Canada; 2 Kidney Research Scientist Core Education and National Training Program, Montreal, QC, Canada; 3 Department of Medicine, Faculty of Medicine & Dentistry, University of Alberta, Edmonton, Canada

**Keywords:** precision medicine, diabetic kidney disease

## Abstract

**Purpose of review:**

Diabetic kidney disease (DKD) is a leading cause of chronic kidney disease (CKD) for which many treatments exist that have been shown to prevent CKD progression and kidney failure. However, DKD is a complex and heterogeneous etiology of CKD with a spectrum of phenotypes and disease trajectories. In this narrative review, we discuss precision medicine approaches to DKD, including genomics, metabolomics, proteomics, and their potential role in the management of diabetes mellitus and DKD. A patient and caregivers of patients with lived experience with CKD were involved in this review.

**Sources of information:**

Original research articles were identified from MEDLINE and Google Scholar using the search terms “diabetes,” “diabetic kidney disease,” “diabetic nephropathy,” “chronic kidney disease,” “kidney failure,” “dialysis,” “nephrology,” “genomics,” “metabolomics,” and “proteomics.”

**Methods:**

A focused review and critical appraisal of existing literature regarding the precision medicine approaches to the diagnosis, prognosis, and treatment of diabetes and DKD framed by a patient partner’s/caregiver’s lived experience.

**Key findings:**

Distinguishing diabetic nephropathy from CKD due to other types of DKD and non-DKD is challenging and typically requires a kidney biopsy for a diagnosis. Biomarkers have been identified to assist with the prediction of the onset and progression of DKD, but they have yet to be incorporated and evaluated relative to clinical standard of care CKD and kidney failure risk prediction tools. Genomics has identified multiple causal genetic variants for neonatal diabetes mellitus and monogenic diabetes of the young that can be used for diagnostic purposes and to specify antiglycemic therapy. Genome-wide-associated studies have identified genes implicated in DKD pathophysiology in the setting of type 1 and 2 diabetes but their translational benefits are lagging beyond polygenetic risk scores. Metabolomics and proteomics have been shown to improve diagnostic accuracy in DKD, have been used to identify novel pathways involved in DKD pathogenesis, and can be used to improve the prediction of CKD progression and kidney failure as well as predict response to DKD therapy.

**Limitations:**

There are a limited number of large, high-quality prospective observational studies and no randomized controlled trials that support the use of precision medicine based approaches to improve clinical outcomes in adults with or at risk of diabetes and DKD. It is unclear which patients may benefit from the clinical use of genomics, metabolomics and proteomics along the spectrum of DKD trajectory.

**Implications:**

Additional research is needed to evaluate the role of the use of precision medicine for DKD management, including diagnosis, differentiation of diabetic nephropathy from other etiologies of DKD and CKD, short-term and long-term risk prognostication kidney outcomes, and the prediction of response to and safety of disease-modifying therapies.

## Abrégé

**Motif de la revue:**

La maladie rénale diabétique (MRD) est une cause majeure d’insuffisance rénale chronique (IRC) pour laquelle il existe de nombreux traitements ayant démontré une capacité à prévenir la progression vers l’insuffisance rénale. Avec son spectre de phénotypes et de trajectoires, la MRD s’avère cependant une étiologie complexe et hétérogène de l’IRC. Dans cette revue narrative, nous discutons des approches de la médecine de précision liées à la MRD—notamment la génomique, la métabolomique et la protéomique—et de leur rôle potentiel dans la prise en charge du diabète et de la MRD. Des soignants et un patient ayant une expérience vécue de l’IRC ont participé à cette revue.

**Sources:**

Les articles de recherche pertinents ont été identifiés dans MEDLINE et Google Scholar à l’aide des termes de recherche suivants: « diabète », « insuffisance rénale diabétique », « néphropathie diabétique », « insuffisance rénale chronique », « insuffisance rénale », « dialyse », « néphrologie », « génomique », « métabolomique » et « protéomique ».

**Méthodologie:**

Revue ciblée et évaluation critique—encadrées par l’expérience vécue d’un partenaire patient et de soignants—de la littérature existante portant sur les approches de la médecine de précision pour le diagnostic, le pronostic et le traitement du diabète et de la MRD.

**Principales observations:**

Il est difficile de distinguer la néphropathie diabétique de l’IRC due ou non à d’autres types de MRD; le diagnostic nécessite généralement une biopsie rénale. Des biomarqueurs ont été identifiés pour aider à prédire l’apparition et la progression de la MRD, mais ceux-ci doivent encore être intégrés aux normes cliniques de soins et évalués par rapport aux outils prédictifs du risque d’IRC et d’insuffisance rénale. La génomique a permis d’identifier plusieurs variantes génétiques causales du diabète néonatal et du diabète monogénique chez l’enfant, lesquelles peuvent être utilisées à des fins diagnostiques et de définition du traitement hypoglycémiant. Des études couvrant l’ensemble du génome ont permis d’identifier des gènes impliqués dans la physiopathologie de la MRD en contexte de diabète de type 1 et 2, mais leurs avantages translationnels restent en deçà des scores de risque polygéniques. Il a été démontré que la métabolomique et la protéomique améliorent la précision diagnostique de la MRD, qu’elles ont été utilisées pour identifier de nouvelles voies impliquées dans la pathogenèse de la MRD et qu’elles peuvent être employées pour améliorer la prédiction de la progression de l’insuffisance rénale, ainsi que pour prédire la réponse au traitement de la MRD.

**Limites:**

Il existe un nombre limité d’études observationnelles prospectives de qualité et d’envergure, dont aucun essai clinique randomisé, appuyant le recours à des d’approches fondées sur la médecine de précision pour améliorer les résultats cliniques des adultes diabétiques ou atteints de MRD, ou présentant un risque pour ces maladies. On ne sait pas exactement quels patients pourraient bénéficier d’une utilisation clinique de la génomique, de la métabolomique et de la protéomique tout au long de la trajectoire de la MRD.

**Conclusion:**

Des recherches supplémentaires sont nécessaires pour mieux évaluer le rôle de la médecine de précision dans la prise en charge de la MRD, notamment pour le diagnostic, la différenciation de la néphropathie diabétique d’autres étiologies de la MRD et de l’IRC, la prédiction des risques à court et à long terme sur le pronostic rénal, et la prédiction de l’innocuité et de la réponse aux traitements pouvant modifier l’évolution de la maladie.

## Introduction

Precision medicine has the potential to provide patients with tailored treatments based on their individual characteristics, including genetic and environmental factors. Examples of precision medicine approaches in nephrology include apolipoprotein L1 (APOL1) risk variants and the treatment of APOL1-associated focal segmental glomerular sclerosis with APOL1 inhibitors^1,2^ and anti-phospholipase A_2_ receptor (anti-PLA_2_R) antibodies for the diagnosis and disease activity monitoring of idiopathic membranous nephropathy.^3,4^ Traditional evidence-based medicine relies on randomized controlled trials (RCTs) to inform treatment decisions, which are designed to include groups of patients rather than individuals. Precision medicine aims to go beyond this by identifying individual patient characteristics that contribute to prognosis or treatment outcomes. The aim of this review is to discuss what is currently known about the clinical landscape of diabetic kidney disease (DKD) as well as precision medicine in DKD with respect to diagnosis, prognosis, and treatment, as highlighted by lived experience from a partner and caregiver of a patient with DKD.

## A Patient Partner/Caregiver’s Journey With DKD

My husband was diagnosed with type 2 diabetes, which we did not take very seriously at first. He did not follow any of the advice given to him by the doctors and nurses and made no real effort to change his lifestyle. This led to many health complications along the way, including the life changing diagnosis of chronic kidney disease. We also did not realize how serious this diagnosis was, and it still did not lead to any efforts to control his diabetes. Things became much more serious 15 years after his diagnosis when he was told he needed to go on dialysis. We were both in shock and I remember thinking we had to live our whole lives in a matter of moments because my husband was now on a rapid track to losing his life. At this point, he finally made the lifestyle changes his care team had been asking him to, but ultimately, it was too late. My husband was on dialysis for 4 years, and this time was filled with many serious health complications, including heart attacks, bypass operations, infections, and blood clots. He passed away at home after a 20-year healthcare journey. As I reflect on this journey, I wonder if my husband had taken better care of his diabetes, would he have delayed the development of chronic kidney disease and would he still be alive today. Living in denial cost my husband his life and cost me my happily ever after story with the person I loved most. I also often wonder if we would have had more precise information about Glen’s risk of developing kidney disease and when he was first diagnosed with diabetes and his risk of kidney failure when he was later diagnosed with kidney disease, if it would have changed his illness and our journey (Arlene Desjarlais, wife of Glen Desjarlais, a patient with diabetes and DKD).

## Objective

To highlight how a patient’s diagnostic and therapeutic journey with DKD can be enhanced through novel metabolomic, proteomic and genomic precision medicine approaches.

## Methods

Original research articles were identified from MEDLINE and Google Scholar using the search terms “diabetes,” “diabetic kidney disease,” “diabetic nephropathy,” “chronic kidney disease,” “kidney failure,” “dialysis,” “nephrology,” “genomics,” “metabolomics,” and “proteomics.” We synthesized the existing literature regarding the precision medicine approaches to the diagnosis, prognosis and treatment of diabetes and DKD framed by a patient partner caregiver’s lived experience. A glossary of terms used through the review is shown in Table 1.

**Table 1.** Glossary of Terms.

Term	Definition
Chronic kidney disease	Long-term evidence of abnormal kidney structure or function including a decline in the kidneys’ capacity to filter the blood, blood or protein in the urine, or imaging abnormalities of the kidneys, such as cysts or scarring
Kidney failure	Advanced kidney disease that requires kidney replacement therapy, which includes dialysis or kidney transplantation
Diabetes mellitus	A disease characterized by a decrease in insulin production by the pancreas or an increase in insulin resistance by organs and tissues that results in elevated blood sugar levels, which may negatively affect small or large blood vessels throughout the body
Diabetic kidney disease	Chronic kidney disease that is in the setting of diabetes mellitus, which may or may not be due to diabetic nephropathy
Diabetic nephropathy	Kidney damage that is directly caused by diabetes mellitus that progresses over years and includes hyperfiltering of the blood, increasing amount of protein in the urine, and a loss of kidney function
Precision medicine	An emerging approach for disease treatment and prevention that takes into account individual variability in genes, environment, and lifestyle for each person
Genomics	The study of all of a person’s genes (the genome), including interactions of those genes with each other and with the person’s environment
Metabolomics	The large-scale study of small molecules, commonly known as metabolites, within cells, biofluids, tissues or organisms. Collectively, these small molecules and their interactions within a biological system are known as the metabolome
Proteomics	The large-scale study of the set of proteins produced in an organism, system, or biological context

## Review

### The Spectrum of Diabetes and DKD

Diabetes mellitus is a metabolic disorder of multiple etiologies, characterized by hyperglycemia due to disturbances of carbohydrate, fat, and protein metabolism originating because of inadequate insulin secretion, insulin action, or both.^5,6^ It is a growing global epidemic with an estimated 536.6 million people currently living with diabetes and this estimate is projected to rise significantly to 783.2 million by 2045.^7^ Uncontrolled diabetes may cause several complications, including coronary artery disease, stroke, chronic kidney disease (CKD) and kidney failure, peripheral vascular disease, retinopathy, and neuropathy.^5,6^ Moreover, the Global Burden of Disease Study 2016 highlighted diabetes mellitus as a leading cause of deaths and disability globally.^8,9^

Diabetic kidney disease is a clinical diagnosis based on decreased estimated glomerular filtration rate (eGFR) and/or the presence of albuminuria in the setting of type 1 or type 2 diabetes.^10^ Diabetic nephropathy is a specific pathological diagnosis defined by glomerular basement membrane thickening, mesangial expansion, and nodularity with the accumulation of extracellular matrix proteins, such as collagen, fibronectin, and laminin, and podocytopathy^11-13^ classified by moderately increased albuminuria (previously microalbuminuria, <30 mg/day), severely increased albuminuria (previously macroalbuminuria, 30-300 mg/day) or overt nephropathy (>300 mg/day). Non-albuminuric DKD is associated with hypertension, dyslipidemia, smoking, and includes classic diabetic glomerulopathy, tubulointerstitial disease, and vascular disease.^14^ Specific pathological classifications for diabetic nephropathy include class I: glomerular basement membrane thickening; class II: mesangial expansion without nodular sclerosis (Kimmelstiel-Wilson lesions) or global > 50% glomerulosclerosis; class III: nodular sclerosis (Kimmelstiel-Wilson lesions); and class IV: advanced diabetic glomerulosclerosis with global >50% glomerulosclerosis with other clinical or pathologic evidence that sclerosis is attributable to diabetic nephropathy.^13^

### Pathophysiology of DKD

Oxidative stress plays a major role in the development of diabetic nephropathy.^15^ Several mechanisms, including auto-oxidation of glucose, advanced glycation end products (AGEs) that act on receptors for AGEs (RAGES), glycolysis, the polyol pathway, glucose-6-phosphate dehydrogenase, the hexosamine pathway, and increased activity of NADPH (nicotinamide adenine dinucleotide phosphate hydrogen) oxidase (Nox) involved in the generation of reactive oxygen species (ROS).^16,17^ Hyperglycemia-induced oxidative stress has been reported to be responsible for the development and progression of diabetic vascular complications including nephropathy by upregulating the transforming growth factor-b1 (TGF-b1) and the extracellular membrane (ECM) protein expression in the glomerular mesangial cells.^18^ Reactive oxygen species are also known to induce TGF-b1 expression, and this further activates stress-activated signaling pathways in diabetic kidney.^19^ In addition, the interaction of TGF-b1 with its receptors induces the phosphorylation and nuclear translocation of the receptor-regulated Smad2 and Smad3 transcription factors. Mesangial cells express Smad2/3 and Smad4, which mediates TGF-b1-induced gene expression, including ECM genes, such as plasminogen activator-1 and type I collagen and fibronectin.^20^ Preclinical and clinical studies using various antioxidants, such as lipoic acid,^21^ vitamin E,^22^ and curcumin,^23^ have been reported to prevent the progression of diabetic nephropathy.

The renin-angiotensin-aldosterone system (RAAS) plays a crucial role in the progression of DKD. Renin breaks down systemic angiotensinogen to Angiotensin I, which then further breaks down to Angiotensin II by the action of angiotensin-converting enzyme (ACE).^24^ Angiotensin II, one of the most important components of RAAS, shows pro-inflammatory, growth stimulatory, fibrogenic, and free radical promoting activities that provoke the development of kidney failure^25^ and diabetic nephropathy^25,26^ mainly acting on Angiotensin II type 1 receptor (AT1R). Angiotensin II also shows most of its pathological effects, such as pro-inflammatory response, hypertrophy, proliferation, and/or apoptosis via AT1R.^27^ Angiotensin II has a prominent role in activation of the nuclear factor kappa B (NF-κB) signaling pathway in diabetic nephropathy. A number of in vivo and in vitro studies show that NF-κB regulates the production of pro-inflammatory cytokines like interleukin-1β^28^ and chemokines, such as MCP-1 (monocyte chemoattractant protein-1), TGF-b1, ICAM1 (intercellular adhesion molecule 1), VCAM (vascular cell adhesion molecule), RANTES (Regulated upon Activation, Normal T cell Expressed, and Secreted), and TNF-α (tumor necrosis factor-alpha).^29,30^ Nuclear factor kappa B activity in macrophages, and glomerular and tubular parenchymal cells, has been linked with parameters of severity of diabetic nephropathy, such as proteinuria, oxidative stress, and inflammation.^31^ Several studies have shown that the elevated levels of Angiotensin II and ACE in patients with diabetes^32^ and blockade of the RAAS were protective in the progression of DKD.^33^

### The Heterogeneity of DKD

Diabetic kidney disease is a complex disease with a wide range of phenotypes. The natural history of diabetic nephropathy (particularly in the setting of type 1 diabetes) was initially thought to be uniformly linear over years with stages that include hyperfiltration, microalbuminuria, macroalbuminuria, overt nephropathy, and subsequent eGFR decline. However, it is now known that the trajectory is variable, ranging from rapid decline in kidney function to remission of initial symptoms of DKD, especially if risk factors are aggressively controlled. Predicting DKD trajectory is challenging due to the variability in individual patient risk factors, co-interventions, and response to therapy. In DKD, the mean rate of eGFR decline is typically 5 to 10 mL/min/1.73 m^2^ per year but is less with standard of care therapy, including blood pressure control, RAAS inhibitors, and sodium-glucose cotransporter-2 (SGLT2) inhibitors, and is dependent on baseline eGFR, albuminuria, and blood pressure.^34-36^ Of course, the structural and functional changes in the kidney as a result of normal aging, which include decreased kidney volume, nephrosclerosis, and reduced nephron number, also influence the rate of eGFR decline as patients with DKD are often older and have had a longer course of diabetes.^37^

Distinguishing diabetic nephropathy from age-related decline can be challenging as clinical factors cannot reliably discriminate between diabetic nephropathy and other DKD and non-DKD phenotypes. For example, a meta-analysis that included 26 studies and 2322 participants showed that independent predictors of non-DKD included the absence of diabetic retinopathy, shorter duration of diabetes, hemoglobin A1c, blood pressure, lipid level, and body mass index, but not age, GFR, or proteinuria.^38^ A kidney biopsy is needed to distinguish DKD from non-DKD, but it is typically not pursued in many settings because of the risks related to percutaneous biopsy and the conclusion that a biopsy is unlikely to change management. A meta-analysis of 48 studies and 4876 patients with diabetes who underwent kidney biopsy identified systolic blood pressure, hemoglobin A1c, diabetes duration, and retinopathy as factors in meta-regression that explained some of the heterogeneity in DKD and non-DKD diagnosis.^39^ A kidney biopsy also cannot reliably identify causal mechanisms of kidney injury if multiple diagnoses are present, which may also be intertwined (eg, DKD and secondary focal segmental glomerulosclerosis from reduced nephron mass). It is unknown how often a kidney biopsy results in a definitive diagnosis of DKD and/or non-DKD across different clinical settings and patient phenotypes but presumably varies by populations and pretest probability.

### Risk Factors of DKD Development, Progression, and Risk Prediction

Traditional modifiable and non-modifiable risk factors for the development of DKD include increased age, male sex, non-European race (eg, Indigenous, persons of color), lower socioeconomic status, obesity, smoking, poorer glycemic control, microvascular complications (retinopathy, neuropathy), and hypertension. Risk factors for the progression of DKD include high blood pressure, acidosis, inflammation, genetics, histology, and blood and urine biomarkers identified in observational studies that classify exposures and adjust for confounders in multivariable regression models. For example, a systematic review of biomarkers (that included 15 studies and 27 biomarkers) predicting onset or progression of DKD in type 2 diabetes beyond conventional risk factors identified the following significant biomarkers: interleukin-18, asymmetric dimethyl arginine, urinary ceruloplasmin, immunoglobulin G, and transferrin for the onset of diabetic nephropathy; asymmetric dimethyl arginine, vascular cell adhesion molecule 1, interleukin-6, von Willebrand factor, and intracellular cell adhesion molecule 1 for the progression of DKD; and high-sensitivity C-reactive protein, E-selectin, tissue-type plasminogen activator, von Willebrand factor, and triglycerides for both.^40^ Whether the adverse outcomes of diabetes that are associated with race and ethnicity are a result of genetic ancestry or systemic racism is a complex issue that needs to be considered in any clinical or research setting of DKD with careful attention to all geographic, sociocultural, and environmental factors.^41,42^

Risk prediction for DKD development and prognosis is important to guide clinical decision-making. This includes screening, interventions, nephrology referral, multidisciplinary CKD care, education, vascular access planning, home dialysis referrals, and kidney transplant assessments.

Risk prediction models for the development of DKD has also been developed and validated. A systematic review of 41 studies including 46 and 18 prognostic models for nephropathy (albuminuria, DKD, CKD, and kidney failure) in type 2 diabetes identified 2 superior models to predict risk for DKD.^43^ The model that included age, body mass index, smoking, diabetic retinopathy, hemoglobin A1c, systolic blood pressure, high-density lipoprotein, triglycerides, and urine albumin to creatinine ratio (ACR) performed the best to predict DKD risk.^44^ Diabetic kidney disease prognosis is typically determined using the 2-year and 5-year kidney failure risk equation (KFRE), which incorporates age, sex, eGFR, and urine ACR^45^ to predict kidney failure and eGFR decline but other risk prediction models for eGFR < 60 mL/min/1.73 m^2^ at 5 years and 40% decline of eGFR at 3 years also exist.^46,47^ Kidney failure risk equation is able to effectively determine the 2-year and 5-year risks of kidney failure, but it predicts higher than observed risk for diabetic nephropathy.^48^ Machine learning algorithms have also been used to predict doubling of serum creatinine and kidney failure with area under the curve (AUC) of >0.80.^49^ Currently, no risk prediction models are perfect and inaccuracy and imprecision may lead to erroneous decisions, most likely related to factors not captured by model inputs, such as blood and/or urine multiomics, which have the potential to improve the accuracy and discrimination of risk prediction models for DKD.^50^

### Treatment of DKD

The treatment of DKD includes blood pressure lowering with lifestyle modification (sodium restriction, weight loss, physical activity, avoidance of smoking, and excess alcohol) and medications (RAAS inhibitors, calcium channel blockers, thiazides with preferential use of other agents for specific indications, such as beta-blockers for coronary artery disease or heart failure),^51^ glycemic control^52^ (hemoglobin A1c < 7%), statins if high cardiovascular risk. RAAS inhibition,^35^ SGLT2 inhibition,^53^ glucagon-like peptide 1 (GLP-1) receptor agonists,^54^ and mineralocorticoid receptor antagonists (MRAs).^55^ However, it remains unclear which individual patients, as opposed to populations of patients generalized from clinical trials, preferentially benefit or are at highest risk of harms of specific interventions, which may be able to be predicted by precision medicine-based approaches.

### Genomics in DKD

One of the most significant progresses toward precision medicine in DKD has been in the treatment of monogenic diabetes. Neonatal diabetes mellitus and monogenic diabetes of the young are the 2 most prevalent diagnoses in monogenic diabetes, and both have had multiple causal genetic variants identified.^56^ Treatments for patients with these conditions can currently be targeted to the specific genetic variant a patient harbors. For example, patients with neonatal diabetes mellitus and a specific methylation signature at the 6q24 chromosomal locus have been shown to respond well to sulphonylureas with regard to glycemic control, while other patients with neonatal diabetes mellitus require treatment with insulin.^57^ Furthermore, sulphonylureas have been shown to be effective treatment in patients with monogenic diabetes of the young caused by mutations in *HNF4A* and *HNF1A* genes, but not in those with mutations in *GCK*, in whom the development of diabetic complications is unlikely.^58^ Monogenic diabetes of the young associated with *HNF1B* mutation requires insulin to achieve adequate glycemic control.^59^ As good glycemic control leads to the prevention and/or delay of the secondary sequelae of diabetes, such as DKD, precision medicine in monogenic diabetes has demonstrated significant success in the individualized treatment of disease. Exome sequencing has been used to improve the diagnosis of monogenetic genetic kidney diseases in the setting of CKD of unknown etiology, such as podocytopathies, collagenopathies, tubulopathies, ciliopathies, but the prevalence of these co-existing disorders in the setting of diabetes is unknown.^60,61^

In contrast to monogenic disease, the translational benefits following from the genetic discoveries in the more common polygenic causes of disease, including type 1 and type 2 diabetes, have been less established. Genome-wide association studies (GWAS) in DKD, conducted in patients with both type 1 and type 2 diabetes, have revealed approximately 33 genes to be associated with disease.^62^ Many of the loci identified help us to understand the pathophysiology of disease, pointing toward the glomerular basement membrane (*COL4A3* and *BMP7*), or to genes implicated in renal biology (*COLEC11* and *DDR1*).^63^ Pathogenetic variants of DKD susceptibility genes identified by whole exome sequencing, such as the COL4A3 gene also partially explained the more severe DKD phenotype (overt proteinuria, rapid progression) in 9 maturity-onset diabetes in young patients with biopsy-proven DKD compared with their parents with less severe DKD.^64^ However, most of these associations have not been replicated in independent diverse cohorts, and the necessary functional work-up of associated genes and specific causal mechanisms have yet to be performed. Furthermore, the associated environmental influences that contribute to disease risk in the setting of complex inheritance are still, for the most part, unknown.

As a first step toward translating these GWAS discoveries toward individualized patient care, a polygenetic risk score for DKD was recently developed.^65^ Polygenetic risk scores are a way to represent an individual’s risk for developing a disease by adding together all the risk loci, weighted by how risky they were found to be in the GWAS analyses. Although the polygenetic risk score provides insight into the distribution of genetic risk for individuals within a population, it does not yet provide clinically applicable results for the individual patient with DKD. Genomic studies also offer promise in identifying novel pathways in DKD pathophysiology and potential targets for drug development, which are desperately needed to improve kidney outcomes.^66^

### Metabolomics and Proteomics in DKD

The metabolome is the global collection of small molecules, typically <1500 Da, which includes carbohydrates, amino acids, organic acids, and lipids.^67^ Related to the metabolome, the proteome is the global collection of proteins that reflect the influence of genetics, epigenetics, and the environment. The metabolome and proteome as they relate to kidney disease are influenced by variation between individuals (as a result of genetics, epigenetics, and the environment),^68,69^ age, comorbidities, GFR,^70-74^ tubular secretion,^75,76^ etiology of CKD,^77^ diet,^78,79^ medications,^80,81^ and gut microbiota.^82-84^ To study the metabolome and proteome, metabolites in bodily fluids are separated using chromatography or electrophoresis followed by high-resolution mass spectrophotometry for untargeted identification and quantification.^85^ Targeted proteomic platforms include Olink and Somalogic. Olink is a proximity extension assay in which oligonucleotide antibody-pairs contain unique DNA sequences allowing hybridization with subsequent proximity extension creating DNA reporter sequences that are amplified by real-time polymerase chain reaction allowing for scalable multiplexing. The Somalogic platform SomaScan consists of protein-capture reagents made of short single-stranded DNA sequences that incorporate hydrophobic modifications that measure native proteins in complex matrices by transforming available binding sites on individual proteins into a corresponding reagent concentration, which is then quantified by hybridization to microarrays. Metabolomics has been used to identify blood and urine metabolites that are associated with adverse clinical outcomes in nephrology, including proteinuria,^86^ incident CKD,^87-89^ CKD progression,^90,91^ kidney failure,^92^ and cardiovascular death.^93^ Proteomic approaches have also been used to evaluate the diagnosis, treatment, and prognosis across a range of etiologies of kidney disease and platforms.^60,94-96^

Metabolomics and proteomics have been evaluated for their ability to improve the accuracy of diagnosis in DKD, to identify novel pathways involved in DKD pathogenesis, and to improve the prediction of CKD progression and kidney failure.^97,98^ For example, the urine proteome measured by liquid chromatography-mass spectroscopy (LC-MS) can accurately distinguish between healthy controls and patients with diabetes (panel of 40 biomarkers, 89% sensitivity, 91% specificity), normoalbuminuria and nephropathy (panel of 65 biomarkers, 97% sensitivity, 97% specificity),^99^ and non-DKD with accuracy (panel of 619 biomarkers with AUC ranging from 0.77 for minimal change disease to 0.95 for vasculitis)^100^ and may be useful in settings where a kidney biopsy is not feasible or its potential risks outweighs its potential benefits. Novel mechanisms of DKD that have been identified by metabolomics/proteomics include innate and adaptive immune responses,^101^ inflammation,^102^ angiogenesis, fibrosis,^103^ tumor necrosis factor pathways, and galactose/glycerol metabolism.^104^

Regarding DKD prognosis and risk prediction, there have been several metabolomic/proteomic studies that have demonstrated conflicting results. In the ORIGIN trial, 15 of 239 biomarkers were independently associated with eGFR change.^105^ In the PROVALID study, a prospective multinational cohort of 2560 adults with type 2 diabetes, a panel of 12 biomarkers explained 34% of variability in DKD progression but the contribution of individual biomarkers was low.^106^ These biomarkers were also unable to discriminate between stable eGFR and rapid eGFR decline beyond baseline eGFR.^107^ In another study of 3 cohorts, 12 biomarkers were associated with >20% decline in eGFR, and 2 biomarkers in particular (kidney injury molecule 1 and beta 2 microglobulin) modestly improved risk prediction in addition to eGFR and albuminuria.^108^ In another study, 13 biomarkers were individually associated with eGFR progression in DKD.^109^ In a case-control study of individuals with type 2 diabetes and an eGFR of 30 to 60 mL/min/1.73 m^2^, 30/207 serum biomarkers were associated with rapid progression of renal failure.^110^ Ongoing efforts, such as the Kidney Precision Medicine Project—a multicenter prospective study in adults with acute kidney injury or CKD who undergo a protocol kidney biopsy—aim to stratify patients based on molecular features of disease, such as histopathology, the transcriptome, the proteome, the metabolome, in addition to clinical characteristics to identify critical cells, pathways, and targets for novel therapies and preventive strategies.^111^

**Figure 1.**
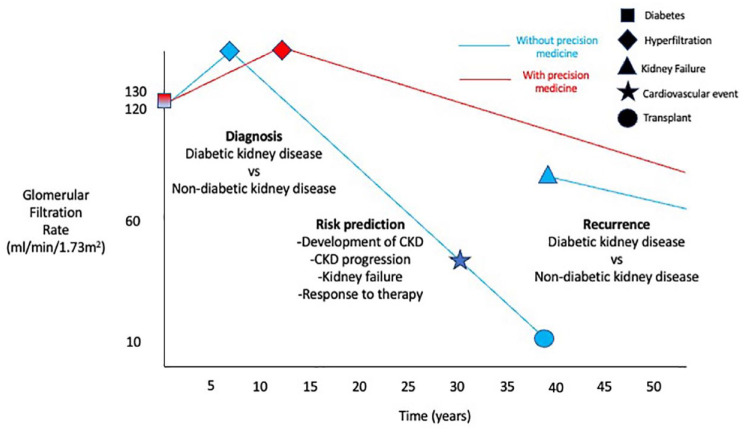
Opportunities for precision medicine using blood and urine metabolomics, proteomics, and genomics across a patient’s journey with diabetic kidney disease to improve clinical outcomes. *Note.* CKD = chronic kidney disease.

Metabolomics and proteomics can also be used to predict response to therapy in DKD. For example, in the PRIORITY trial, the urine CKD273 classifier predicted progression to microalbuminuria, CKD and 30% decrease in eGFR,^112^ as well as the response to spironolactone.^113^ In addition, urinary polypeptides identified in this trial predict responsiveness to angiotensin receptor blockers.^114^ Systems biology approaches could be used in future trials of DKD to select for high-risk participants to enrich the trial population for those at the highest risk of events to reduce the sample size requirement beyond eGFR and ACR eligibility criteria.^115^ Figure 1 summarizes the potential opportunities for blood and urine metabolomics, proteomics, and genomics across a patient’s journey with DKD, including are more accurate and timely diagnosis, better risk prediction and timely initiation of targeted therapies to prevent CKD progression, kidney failure, and other complications. Biomarker-adaptive designs have been used in RCTs in oncology to phenotype participants at baseline for adaptive randomization to maximize treatment responses and statistical efficiency. In the I-SPY2 adaptive RCTs, adults with stage 2 or 3 breast cancer with tumors >2.5 cm were phenotyped into 8 biomarker subtypes based on herceptin 2, hormone receptors (estrogen, progesterone) and a 70 gene assay to determine responses to different chemotherapy regimens.^116,117^ These novel approaches are lagging in nephrology RCTs but are promising although they require significant biostatistical expertise.^118^

## Conclusion

In summary, DKD is a heterogeneous disease with a wide range of clinical presentations, biomarker/genetic profiles, treatments, and outcomes. Improvements in understanding disease pathophysiology and individualizing treatments are needed. Though the results of the reported genetic and metabolomic/proteomic studies are representative of a model for using genomics and metabolomics/proteomics in precision medicine of DKD, we still have far to go. At present, we do not yet have the tools to truly implement precision medicine in DKD. In the future, stratifying clinical research cohorts by relevant high-risk and low-risk genotypes or metabolomic/proteomic profiles may help to make clinical trials in DKD more effective, and in turn, lead to individualized treatment protocols and improved outcomes, experiences, and quality of life for patients. Perhaps in the future, precision medicine will be used to assist patients, caregivers and clinicians (like Glen, Arlene, and their circle of care) to have a better understanding of the risks of both diabetes and DKD and can better inform shared decision-making to initiate lifestyle interventions and pharmacotherapy to improve clinical outcomes. Finally, clinicians, researchers, funding agencies, and policy-makers must strive to ensure that precision medicine approaches are inclusive and equitably accessible to diverse groups of patients to maximize individual and population outcomes.^119^
